# Event and Comment

**Published:** 1911-07-15

**Authors:** 


### DENTAL REGISTER.

**Vol. LXV. July 15, 1911. No. 7**

### EVENT AND COMMENT.

PREVENTION OF CARIES. At no time in the history or the medical or dental professions has there been so much thought and effort given to the problem of preventive medication as at the present. One can hardly pick up a medical magazine at random without encountering an article on the subject, and even the popular magazines and newspapers are taking up the subject too often, however, in a more optimistic spirit than medical science warrants. How much of this popular agitation is based on the wide spread mental science healing and faith cure propaganda, and how much on the great discoveries of medical science of recent years it is of course difficult to estimate. It is, nevertheless, here and must be reckoned with. In dentistry its most aggressive manifestation is seen in the tremendous efforts being made to introduce dental hygiene into the public schools not only as a part of the curriculum, but as a sanitary measure with legal enforcement to insure protection from infective and contagious diseases. We do not want to discourage in any way this public work, as it can do no possible harm, unless it should pass away so quickly that no permanent benefits shall come from the agitation, but we believe that the public mind is now in a condition to be moulded into right thinking and profitable action. And to help on the work by giving out a permanent basis for education of the people through the dental profession, who should be the teachers and leaders we are giving up a large amount of space in this issue to reprint from the *Dental Record*, of

England, an article on "The Prevention of Dental Caries" by Dr. Stanley Colyer, which gives the most complete summary of our knowledge of the etiology of caries and its treatment that we have ever seen. We believe that it will be worth the while of every dentist to read this article carefully and get from it the main points that he may communicate them to his patients as he comes in contact with them and has opportunity. We tried to make an abstract of the paper, but became convinced that this would not meet the needs of the profession. The timeliness of the subject and value of the paper must stand as our reason for giving up so much space to the paper. It is a long paper, but it is full of meat and will pay any one for even a cursory inspection.

NATIONAL DENTAL ASSOCIATION. By the time this issue reaches its readers our national organizations will be in session in Cleveland. The dentists of Cleveland are making strenuous efforts to secure and entertain the largest gathering of this body ever held. Because of the central location and the many important national organizations called to meet at this place and time, there is every probability that this will be more largely attended than any other meeting of that body. A fine programme is offered and the proceedings will be full of interest. We hope to present in the August issue a brief report of the doings of the several organizations, but of course, it will be much better for one to be on hand to get complete impressions. It is highly desirable that all who are interested in the reorganization of the Association be present with such practical ideas as they may have, that all phases of the subject may be presented, as much depends on the way in which this matter is settled. The oral hygiene matter must also be carefully considered at this time, if the results of the work already done are to be utilized and moulded into some practicable form of preventive dentistry. The agitation period has been well done, but the hardest part of the problem is still to be solved. We must now make good our theories.

Another important matter to come before this meeting is the report of the Committee on Law and Education. This will probably not be a finished report, as the subject is one that can not be arbitrarily settled by any action of even the National Dental Association; but it is hoped that by continued agitation some more uniform standards than we now have may be attained.

